# The comparisons of inhibitory control and post-error behaviors between different types of athletes and physically inactive adults

**DOI:** 10.1371/journal.pone.0256272

**Published:** 2021-08-16

**Authors:** Chia-Chuan Yu, Neil G. Muggleton, Chiao-Yun Chen, Cheng-Hung Ko, Suyen Liu

**Affiliations:** 1 Department of Kinesiology and Health Education, The University of Texas at Austin, Austin, Texas, United States of America; 2 Institute of Cognitive Neuroscience, National Central University, Taoyuan City, Taiwan; 3 Institute of Cognitive Neuroscience, University College London, London, United Kingdom; 4 Department of Psychology, Goldsmiths, University of London, London, United Kingdom; 5 Department and Graduate Institute of Criminology, National Chung Cheng University, Chiayi, Taiwan; 6 Integrated Drug Addiction Treatment Center of the Jianan Psychiatric Center, Ministry of Health and Welfare in Taiwan, Tainan, Taiwan; 7 Department of Athletic Sports, National Chung Cheng University, Chiayi, Taiwan; University of Tsukuba, JAPAN

## Abstract

To properly behave and correct mistakes, individuals must inhibit inappropriate actions and detect errors for future behavioral adjustment. Increasing evidence has demonstrated that athletes are superior in cognitive functions and this benefit varied dependent on the types of sport that individuals involved in, but less is known on whether athletes have a different error-related behavioral pattern. The purpose of this study was to compare the behavioral performance of inhibition and error monitoring between individuals who participated in an open-skill sport (n = 12), a closed-skill sport (n = 12), and a sedentary lifestyle (n = 16). A combined flanker/stop signal task was presented and the derived stop signal reaction time (SSRT), post-correct accuracy and reaction time (RT), as well as post-error accuracy and RT were compared across groups. Our findings indicated there was no difference in SSRT between groups. Surprisingly, significant post-error slowing (PES) was observed only in controls but not in sport groups, the controls also exhibited significantly longer post-error RT compared with the open-skill group. However, there was no difference in the post-error accuracy between groups, indicating a higher efficiency in the post-error processing among open- and closed-skill groups by requiring comparatively less time for behavioral adjustments. The present study is the first to disclose the discrepancies in PES between different types of athletes and controls. The findings suggest that sport training along with higher amounts of physical activity is associated with a more efficient behavioral pattern for error processing especially when the sport requires open skills in nature.

## Introduction

There are a large number of situations that demand the cognitive process of withdrawing improper actions (e.g. hitting brakes when an individual sees the red light while driving) which is also known as inhibitory control [1], a basic element of executive function that facilitates self-regulation [2] and associated with the activation in the prefrontal cortex [3]. Such ability could be predictive of cognitive and social competencies [4], whereas inhibitory deficits are related to problematic behaviors such as violence [5], heavy alcoholic drinking [6] as well as impulsivity [7].

Once individuals fail to stop an ongoing behavior, they may be aware of the error and adjust further actions (i.e. error monitoring) to ensure better performance in the future [8], resulting in slower responses in the subsequent trials, a phenomenon termed post-error slowing (PES) [9] which is a reliable effect within individuals [10]. This information-processing leads to not only the awareness of a mistake but also influences the learning process [11] to adapt behaviors to meet the requirements in a given task. Moreover, this cognitive ability is operated depending on the perceived feedback [12] and types of error [13], which are reinforcement learning stimuli to trigger the dopamine system and anterior cingulate cortex [14]. Empirical evidence has shown the relationships between aberrant inhibition, diminished PES, and clinical symptoms.

Interestingly, event-related potentials (ERPs) studies demonstrated that participation in exercise is beneficial for cognitive function including action monitoring [15]. Themanson and Hillman [16] indicated individuals with a higher fitness level demonstrated longer PES, decreased error-related negativity (ERN), and increased error positivity (Pe) amplitudes than less-fit individuals, suggesting enhanced attention control in the error-related processing to provide more effective behavioral adjustment [16]. In a later study, higher fitness was linked to increased post-error accuracy and ERN. Notably, although the findings of these two studies seem to be inconsistent regarding the ERN, the fitter participants in the latter study could be predictive of more corrections after errors which means fitness modulated the adaptability for improving further behaviors via more cortical activation in the network of error processing [17], while the results of the former study may reflect the ability of error awareness without as much cognitive effort as required by the lower-fitness group. Similarly, a study examined the differences in inhibition and error detection among the elders who regularly participated in open- or closed-skill exercises or erratically participated in exercise, found shorter reaction time (RT) for both regular exercise groups, the open-skill group revealed reduced N2 and increased P3a amplitudes in a Stroop task compared with controls. Additionally, shorter ERN latency showed in the open-skill group than in controls at a task-switching task [18], indicating not only improved neural efficiency for error monitoring among open-skill individuals but also supporting superior inhibitory processing in both sport groups.

An increasing number of studies support the notion that chronic exercise participation [19] is correlated with the improvement of inhibition via the potential mechanisms of increased brain structure/functioning, including higher volume of the dorsal striatum [20], greater activation in prefrontal and parietal areas [21]. Indeed, trained athletes performed better cognitive control, such as open-skill sport athletes revealed shorter stop signal reaction time (SSRT) than closed-skill sport athletes and controls [22]; fencers showed a lower error rate, decreased RT, greater N2, and smaller P3 amplitude in a go/nogo task [23, 24]; volleyball athletes performed decreased SSRT and increased successful inhibition [25]; as well as elite soccer players who demonstrated descended SSRT compared to non-elite players and non-athletes [26]. Moreover, athletic training may not merely link to better behavioral performance, Di Russo, Taddei, Apnile, and Spinelli’s work [27] found shorter RT in a discriminative response task among fencers. Further, these fencers exhibited larger N1, N2, and P3 amplitudes as well as estimated stronger activation in the anterior cingulate gyrus according to the source analysis, suggesting improvement in early visual and inhibitory processing. However, although extensive evidence uncovered the beneficial role of sports on inhibitory control, literature questioned whether they are also associated with the behaviors regarding error monitoring is relatively impoverished. Thus, the present study is the first aims to investigate not only the behavioral difference in inhibition but also the possible modulation of post-error adjustment between dichotomous types of athletes and individuals with a sedentary lifestyle.

Overall, the typology of sports as being either closed-skill or open-skill, the former one refers to the sports with an absence of direct interaction with an opponent or the environment and usually occur in a self-paced context, thus, little adjustment based on unpredictability is required for athletes (e.g. swimming). In contrast, the open-skill sport has relatively frequent changes including the dynamic interactions with adversaries, it has lower predictability and an associated reduction in the knowledge of which actions to be prepared ahead of time (e.g. taekwondo). Previous studies compared the cognitive capacities between individuals who participated in closed- and open-skill sports, including visuospatial attention and memory [28], simple reaction and discriminative reaction [29], inhibition and/or error-related processing [18, 22], visuospatial attention and inhibition [30], and so on. Although it has been reported that higher fitness is associated with greater PES [16] and benefits for action monitoring processes [17], there is currently no study that we are aware of that has investigated whether PES is different for groups engaged in different types of sport.

The current study, therefore, applied a combined flanker/stop-signal task to compare the SSRT and PES between open-skill sport athletes, closed-skill sport athletes, and sedentary controls. We chose taekwondo as the open-skill sport due to its abundant opportunities for interaction with external stimuli and requiring a faster response, higher degree of cognitive flexibility and attention. In addition, experimental studies indicated that chronic intervention of taekwondo enhances a variety of cognitive function such as information processing speed [31] and executive function [32] among people in a wide range of age, which might due to the increased neurotrophic growth factors such as brain-derived neurotrophic factor (BDNF) and insulin-like growth factor-1 (IGF-1) [33], these are crucial elements for cognitive development and brain plasticity. On the other hand, swimming was selected as the closed-skill sport because of its relatively constant characteristics and little to no interaction with the environment which may subsequently contribute to less improvement in cognitive function. Nonetheless, research has proven that long-term water aerobic exercise improves cognitive outcomes including executive function [34], inhibition [35], attention and cognitive flexibility [36]. Furthermore, cross-sectional research showed the relationship between regular swimming and superior executive function among older adults [37]. The sedentary controls were those who have no specialty in sport and keep a physically inactive lifestyle in the past five years before and during the current study.

The aforementioned design allowed testing for the following hypotheses. First, it was predicted that inhibitory control would be best in the open-skill sport group (i.e. shortest SSRT) compared with the other two groups while the closed-skill group would not significantly differ from controls, this would be consistent with the idea that open-skill sport athlete may outperform than closed-skill sport athletes and non-athletes as the previous study showed [22]. Second, it was predicted that PES would be modulated by expertise in sports, two sport groups would show increased PES compared to controls, consistent with the findings of Themanson and Hillman [16], which regards higher amounts of physical activity (PA) could potentially lead to better fitness that was associated with greater PES.

## Materials and methods

The Human Research Ethics Committee at National Chung Cheng University in Taiwan approved all study procedures and all participants gave informed consent prior to participating in this study, which was conducted in accordance with the Declaration of Helsinki. We utilized a cross-sectional design with three separate groups. Outcome variables included measures of Go accuracy, Go RT, Stop accuracy, SSRT, post-correct accuracy/RT, along with a measure of post-error accuracy and speed.

### Participants

Forty-two university students were recruited in this study using online advertisements, including 13 taekwondo athletes for the open-skill sport group and 12 swimmers who formed the closed-skill sport group. The training programs for both athletic groups consisted of at least three hours per day for three or more days per week and that they have been trained for at least two years. The athletes in the current study were all qualified for the first class of National Intercollegiate Athletic Games in Taiwan which is one of the highest levels of national competitions for taekwondo and swimming varsities in Taiwan. Seventeen non-athletes formed the sedentary control group, they had no expertise in any sport and had a sedentary lifestyle in the past five years and during the study, which was defined as taking part in exercise no more than once per week and for no more than 2.5 hours on each occasion. We excluded two participants who had a history of brain injury (one from the open-skill group and the other one from the controls), remaining total of 40 participants which are 12, 12, and 16 participants in the open-skill (4 males), closed-skill (6 males), and sedentary control groups (4 males) respectively. All participants had no history of any neurological disorder and all had a normal or corrected-to-normal vision.

### Study procedure

After signing the informed consent form, all participants conducted two parts of the current study. The demographic data, amounts of PA, and length of training experience (months) were collected in the first part while a cognitive task was performed in the second part. After the cognitive assessment, each participant was fully debriefed about the study aim. As Faul, Erdfelder, Lang, et al. [38] recommended, a priori power analysis was applied using G*Power 3.1 software to determine the sufficient sample size for detection of the difference in cognitive performance. The estimation was calculated based on an expected medium correlation between 4 repeated measures (*r* = 0.5) and medium effect size (*f* = 0.25) for the interplay between factors [39, 40], the power analysis indicated that 10 participants in each group are needed to achieve 80% statistical power at α = 0.05.

### Assessment of physical activity

The amounts of PA for all participants were assessed using the Taiwan version of the International Physical Activity Questionnaire long version (IPAQ long-version) to compute the total metabolic equivalent task in minutes per week (total MET-minutes/week). This questionnaire required recall of PA over the preceding seven days and has been shown to have a good content validity [41].

### Task design and procedure

The experiment took place in a sound-attenuated room using a laptop with a 14-inch screen of resolution 1366 x 768 pixels and a vertical refresh rate of 60 Hz. Participants sat comfortably 57 cm in front of the screen which was at eye level. The task was presented using E-Prime 2.0 software (Psychology Software Tools, Pittsburgh, PA).

**Fig 1:**
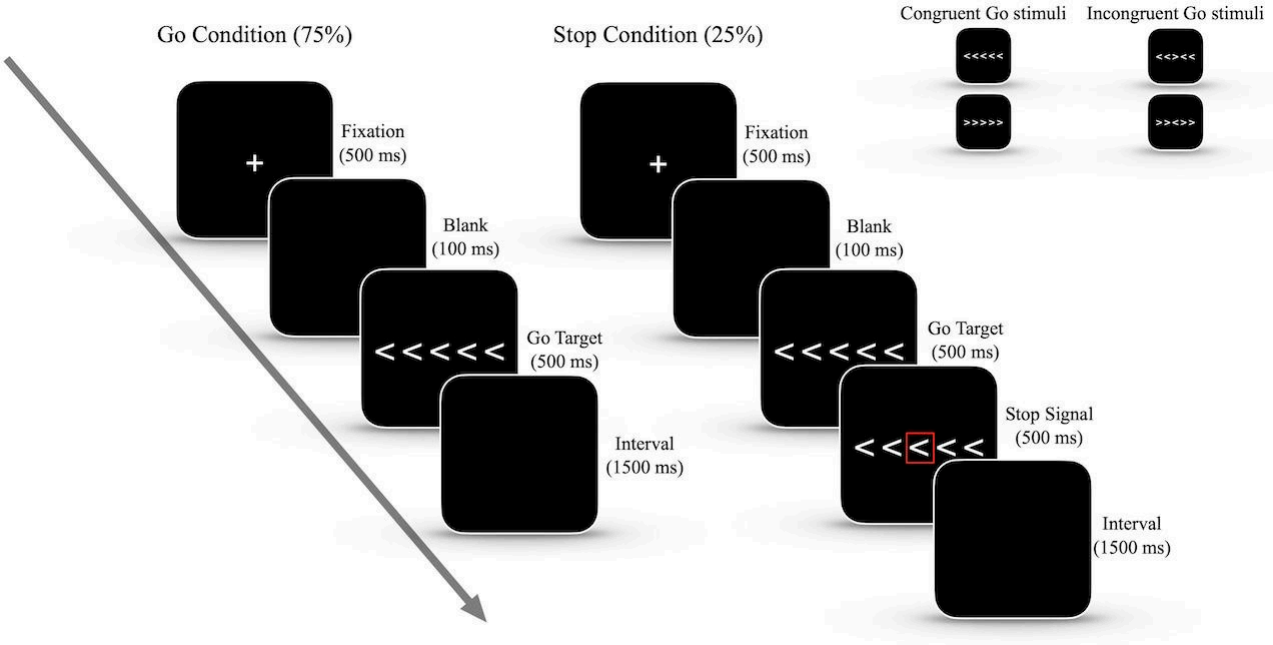
The time-line for a trial of the flanker/stop signal task.

The flanker/stop signal task (Fig 1) involved the presentation of a fixation cross (500 ms) followed by a brief blank (100 ms) and then the presentation of the target (go stimulus) to which a response was required. The targets consisted of five arrows, with the correct response being to indicate the direction of the central element. For the left- or right-pointing target, a press of the “D” or “L” key with the left/right index finger had to be made on the keyboard. The surrounding elements were either all pointing in the same direction as the central element for congruent trials (>>>>> or <<<<<) or were in the opposite direction to the central element for incongruent trials (e.g. >><>> or <<><<). A total of 480 trials were presented with equal probabilities for pointing left/right and congruency/incongruency.

Participants were instructed to respond to the go stimulus as quickly and accurately as possible. On 25% of the trials a stop signal (i.e. a red box) was presented after the onset of go stimulus, which was initially presented with a latency of 200 ms (i.e. stop signal delay, SSD), the successful inhibition required the response to the go stimulus to be withheld. After each stop trial, the SSD was adjusted based on the accuracy with the aim of the SSD producing a successful stopping rate at about 50%. If subjects were unable to successfully withhold a response, the SSD was reduced by 34 ms for the next stop trial, whereas a success led to an increase of the SSD by 34 ms.

The behavioral performance was calculated for both congruent and incongruent conditions as followed: *1) Go accuracy*: the percentage of go trials where a correct response was made; *2) Go reaction time (Go RT)*: the average time that was taken to correctly respond on go trials; *3) Stop error rate*: the percentage of stop trials where the response was not canceled; *4) Stop signal reaction time (SSRT)*: a behavioral index that represents the time needed for the inhibitory processing, this was calculated based on the race model [1] and employed the distribution of Go RTs for each individual in combination with the probability of successfully stopping on stop trials for different SSDs [42, 43]; *5) Post-correct accuracy and RT*: the mean accuracy and RT for the go trials performed immediately after a correct go trial; 6) *Post-error accuracy and RT*: the mean accuracy and RT for go trials presented immediately after an incorrect stop trial. Data was excluded if the go trial: 1) have a RT which exceeds 3 SD of overall data; or 2) without response; or 3) pressing with the wrong direction.

### Statistical analysis

All analyses were conducted using the IBM SPSS 26.0 software with the significant level set at *p* ≤ 0.05. The age, education, height, weight, body mass index (BMI), and the amount of PA for three groups were compared using a one-way analysis of variance (ANOVA) and Tukey HSD approach to further examine any significant differences. Due to the amounts of PA were missing for some participants in the control group (n = 4, 10%), we applied an expectation-maximization approach to treat the missing data [44] which is a method with less bias. The length of training experiences (months) of open-skill and closed-skill sport groups were compared by using a two-tailed independent *t*-test. To compare the performance of Go accuracy, Go RT, stop error rate, and SSRT, we used a two-way [3 (groups) x 2 (conditions: congruent or incongruent)] mixed ANOVA with a Bonferroni adjustment for further comparisons. We used a three-way [3 (groups) x 2 (conditions) x 2 (correctness: post-correct or post-error trials)] mixed ANOVA to compare all post-error behaviors with a Bonferroni adjustment for further comparisons.

## Results

### Demographics

The demographic data are shown in Table 1, analyses revealed no significant difference among groups for age, weight, height, BMI, or education (all *p*s > 0.05), but the IPAQ data showed differences between groups (*F*_(2, 37)_ = 7.91, *p* = 0.001, η_p_^2^ = 0.30), comparisons of means using the Bonferroni approach indicated that both open-skill (*p* < 0.01) and closed-skill sport groups (*p* < 0.05) have a significantly higher amount of PA than controls while the two sport groups were not significantly different from each other. There was no difference of training experience across sport groups (*t*_(22)_ = 0.96, *p* = 0.35).

**Table 1:** Demographic statistics of the open-skill sport, closed-skill sport, and control groups.

Group Variable	Open-skill sport (n = 12)	Closed-skill sport (n = 12)	Sedentary control (n = 16)	
	M (SD)	M (SD)	M (SD)	*F*
Age (years)	21.00 (1.13)	20.83 (1.34)	20.31 (0.60)	1.74
Weight (kg)	65.75 (16.30)	65.92 (14.40)	57.50 (12.85)	1.61
Height (M)	1.68 (0.10)	1.69 (0.09)	1.65 (0.11)	0.82
BMI (kg/m^2^)	23.12 (4.70)	22.75 (3.03)	20.96 (2.63)	1.58
Education (years)	14.58 (1.31)	14.58 (1.44)	14.94 (0.93)	0.41
IPAQ (total MET-minutes/week)	17821.54 (6769.84)	14297.85 (15606.75)	3926.93 (4440.83)	7.91***
	M (SD)	M (SD)		*t*
Training (months)	148.75 (30.53)	132.50 (49.84)	–	0.96

Note

*** represents the *p*-value < 0.001;—refers to have no experience of athletic training in the control group.

### Go response

The descriptive statistics for all behavioral performance are shown in Table 2. Analyses showed a significant main effect of condition (*F*_(1, 37)_ = 70.32, *p* < 0.001, η_p_^2^ = 0.66), with higher Go accuracy in the congruent condition than in the incongruent condition. There was no significant effect of group (*F*_(2, 37)_ = 0.34, *p* = 0.72), nor a significant interaction (*F*_(2, 37)_ = 2.15, *p* = 0.13). Similarly, a significant main effect of condition (*F*_(1, 37)_ = 306.83, *p* < 0.001, η_p_^2^ = 0.89) was found for Go RT, with longer RT in the incongruent condition than in the congruent condition. There was neither significant effect of group (*F*_(2, 37)_ = 1.68, *p* = 0.20) nor a significant interaction (*F*_(2, 37)_ = 1.25, *p* = 0.30).

**Table 2:** Descriptive statistics of behavioral performance across groups and conditions.

Group Variable Condition		Open-skill sport (n = 12)	Closed-skill sport (n = 12)	Sedentary control (n = 16)
		M (SD)	M (SD)	M (SD)
Congruent	Go accuracy	0.99 (0.01)	0.96 (0.06)	0.99 (0.01)
	Go RT (ms)	369.81 (25.48)	394.21 (44.67)	400.00 (36.28)
	Stop error rate	0.64 (0.09)	0.64 (0.05)	0.62 (0.07)
	SSRT (ms)	229.28 (30.56)	224.18 (38.56)	232.70 (30.65)
	Post-correct RT (ms)	374.19 (36.67)	397.60 (56.61)	410.13 (48.04)
	Post-error RT (ms)	380.69 (37.59)	410.82 (68.73)	448.75 (62.97)
	Post-correct accuracy	0.99 (0.02)	0.98 (0.05)	0.99 (0.02)
	Post-error accuracy	0.98 (0.03)	0.96 (0.09)	0.98 (0.06)
Incongruent	Go accuracy	0.86 (0.08)	0.90 (0.09)	0.89 (0.08)
	Go RT (ms)	425.67 (35.96)	442.58 (46.29)	445.24 (36.94)
	Stop error rate	0.38 (0.11)	0.40 (0.07)	0.39 (0.07)
	SSRT (ms)	270.14 (35.08)	255.64 (45.10)	257.87 (36.83)
	Post-correct RT (ms)	436.76 (48.62)	455.20 (56.98)	464.76 (54.29)
	Post-error RT (ms)	452.92 (61.58)	452.67 (44.60)	487.19 (54.70)
	Post-correct accuracy	0.88 (0.07)	0.88 (0.10)	0.89 (0.11)
	Post-error accuracy	0.82 (0.17)	0.85 (0.14)	0.86 (0.12)

### Inhibitory control

The analysis of stop error rate showed there was a main effect of condition (*F*_(1, 37)_ = 115.14, *p* < 0.001, η_p_^2^ = 0.76), with more errors for the congruent condition than for the incongruent condition. There was no significant effect of group (*F*_(2, 37)_ = 0.39, *p* = 0.68), nor a significant interaction (*F*_(2, 37)_ = 0.12, *p* = 0.89). With regard to SSRT, there was a main effect for condition (*F*_(1,37)_ = 128.85, *p* < 0.001, η_p_^2^ = 0.78) with shorter SSRT in the congruent condition. There was no significant effect for group (*F*_(2, 37)_ = 0.24, *p* = 0.79), nor a significant interaction (*F*_(2,37)_ = 2.62, *p* = 0.09).

### Post-error behaviors

**Fig 2:**
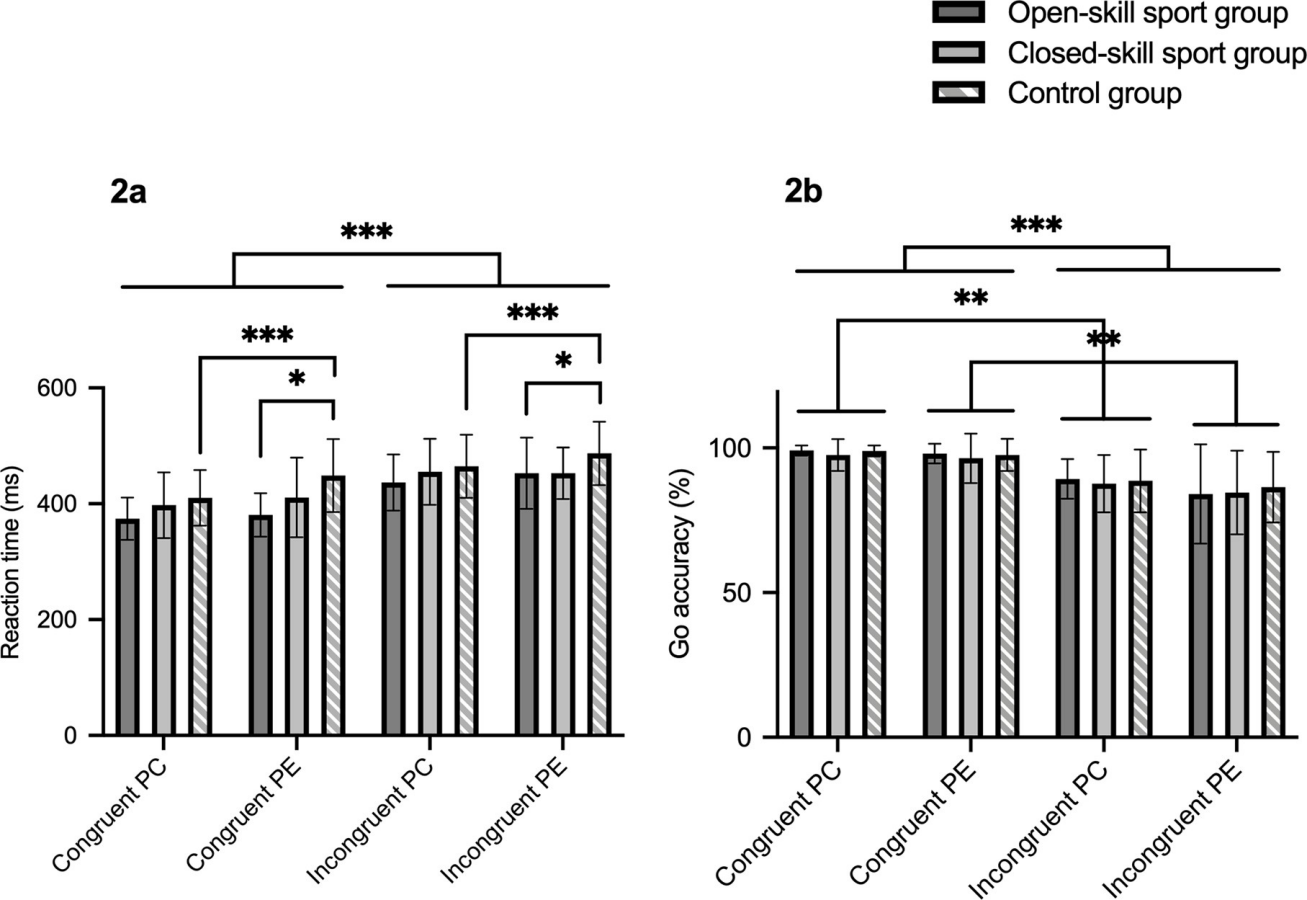
Mean and SD of (a) post-correct and post-error RTs and (b) post-correct and post-error accuracies across congruent and incongruent conditions for open-skill sport group, closed-skill group, and control group; PC = post-correct; PE = post-error; *** represents the *p*-value ≤ 0.001; ** denotes the *p*-value ≤ 0.01; * refers to the *p*-value ≤ 0.05.

Fig 2 demonstrates the post-correct and post-error accuracies/RTs, the analyses of RTs revealed a significant main effect for condition (F_(1, 37)_ = 133.99, *p* < 0.001, η_p_^2^ = 0.78) with shorter RT in congruent condition, and a significant effect of correctness (F_(1, 37)_ = 17.62, *p* < 0.001, η_p_^2^ = 0.32) with there being slower RT in the post-error trials than in the post-correct trials while the effect of group was not significant (F_(2, 37)_ = 2.52, *p* < 0.10). Nevertheless, a significant interaction between correctness and group was observed (F_(2,37)_ = 4.48, *p* = 0.02, η_p_^2^ = 0.20), simple effects showed that the significant phenomenon of PES (i.e. significantly prolonged RT after errors compared with after correct reactions) was only for the control group (*p* < 0.001) but not for sport groups (*p*s > 0.05). Furthermore, the sedentary controls exhibited increased RT than the open-skill sport group in the post-error trials (*p* = 0.04), whereas neither difference between sport groups in the post-error trials nor difference between three groups in the post-correct trials were found (*p*s > 0.05).

For the accuracies following correct and error trials, there were significant main effects for condition (F_(1, 37)_ = 43.68, *p* < 0.001, η_p_^2^ = 0.54) and correctness (F_(1, 37)_ = 6.65, *p* = 0.01, η_p_^2^ = 0.15), subjects exhibited higher accuracy in congruent condition as well as decreased accuracy in post-error trials. The analysis showed that the main effect of group and the interaction between factors were insignificant (*p*s > 0.05).

## Discussion

The growing literature suggested that individuals who undergo chronic sport or exercise possess superior cognitive capabilities including inhibition and error monitoring compared with their counterparts who take part in lower amounts of physical activity. Meanwhile, these effects varied depending on which types of the sport were taken, with the example of enhanced inhibitory control seen for individuals who engaged in the interceptive sport but not for closed-skill sport athletes [22], or the elders who regularly participated in open-skill exercise showed faster processing for error monitoring than the irregular exercisers [18]. However, literature was lacking to investigate whether the beneficial role of sport also occurs for the error-related behavioral patterns as well as whether the type of sport is a factor that modulates the PES. Thus, this study was the first to demonstrate the difference in the pattern of PES among open-skill sport (taekwondo) athletes, closed-skill (swimming) athletes, and sedentary controls, whereas there was no significant modulation in inhibitory control between groups.

Participants were grouped based on their athletic experience, both sport groups reported larger amounts of PA than the sedentary control group while there was no significant difference between sport groups. To examine our hypotheses, the flankers and infrequent stop signals were adopted, which required participants to ignore irrelevant information and to inhibit prepotent behaviors. In addition to inducing the inhibitory control, the SSD was adjusted to maintain the stop error rate at about 50%, which allows us to ensure adequate post-error trials for analyses. Consistent with previous studies that used interferential stimuli, we found a main effect of flankers that participants performed lower accuracy with slower speed when facing incongruency [e.g. 36, 45].

Unlike previous studies, our SSRT data rejected our first hypothesis which possibly due to the cognitive task in the current study. Although we united a diversity of components from the flanker task and stop signal paradigm to evaluate the potential modulation of sport groups, the change in inhibitory control might require a more sport-related task design, a requirement seen for the effects on cognition elsewhere [46]. Any modulation on inhibitory control associated with sport training may not transfer to the task used here, even though the discipline they underwent might correlate with improved cognition. Perhaps the effects on inhibition in previous studies cannot be completely generalizable for transfer between paradigms or different settings, the transfer of benefits to a situation where the learned sport cues (e.g. timer and opponents) are absent would not be likely.

Another latent reason could be that the health risks among the open-skill sport individuals, in contrast to the participants in previous studies such as tennis varsity and fencers [22, 24], taekwondo is comparatively risky for having sport-related concussion due to its abundance of impacts [47]. However, though the inclusion criteria we set have addressed the confounding factors regarding brain injury, this possible under-reporting and the essence of taekwondo might still blur our results by offsetting the beneficial role of open-skill sport. Exposure to sport- or recreation-related concussion is associated with the alteration of the primary motor cortex [48] and attentional deficits [49] which are vital in the network of response inhibition [50, 51].

The current assessment of post-error behaviors is the first to demonstrate the differences in the pattern of PES between athletes and non-athletes. Specifically, the significant PES was only observed for the controls but not for the athletic groups. Furthermore, the open-skill sport group showed decreased post-error RT compared to the control group, while there was no difference between sport groups or between swimmers and controls. Although the current data also rejected our second hypothesis, this might not reflect the impaired error-related cognition among athletes. Instead, the sport groups showed more effective error processing that was reflected by the insignificant post-error accuracy between groups. The open-skill sport participants performed a more efficient behavioral mode by taking less time to succeed in the upcoming adjustment while the control group demanded more time to achieve equivalent performance. The varying degrees of PES based on the sorts of the sport were also available, in spite of the absent difference between the closed-skill sport group and controls, the closed-skill sport individuals had a similar pattern as the open-skill sport group showed (i.e. an insignificant PES). Therefore, our findings support the psychophysiological evidence of Li et al. [18] which revealed shorter latency of ERN in the open-skill group, reflects the efficacy of the network that relies on the basal ganglia to the anterior cingulate cortex for the error awareness.

Furthermore, the current study seems to contradict with the previous findings that greater PES among people with better fitness [16] and the associations between fitness and increased post-error accuracy [17] which suggested a higher fitness level is correlated with increased error awareness and the modulation of its neural processing, whereas our results indicated that both sport groups demonstrated more efficiency for detecting and adjusting post-error behaviors independent of congruent conditions, possibly attributed to the combination of greater PA and regular sport training especially when the sport required greater cognitive demands (i.e. open-skill sport).

Additionally, the discrepancies of the procedure between cognitive tasks and the psychological characteristics of participants might also contribute to our PES findings. First, although the samples in the previous studies [16, 17] possessed higher fitness performance, they were not necessarily athletes, thus these individuals may not experience any systematic sport training that is putatively thought to be associated with abundant cognitive stimuli in routine. Secondly, Themanson et al. [17] attested the effects that the instruction of cognitive assessment being accuracy- or speed-emphasized could affect the behavioral performance of error processing. Remarkably, the increased PES among fitter individuals that Themanson and Hillman [16] found was in a speed-stressed condition. In the current study, however, we accented both accuracy and speed for administrating the task rather than using only one of the instructions of previous studies (i.e. either accuracy- or speed-emphasized conditions), perhaps the different settings could lead to varied inhibitory and error processing strategies. Third, in relation to the percentage of correct responses, previous studies showed relatively higher accuracy between 79–90% in different conditions [16, 17] compared with our accuracy that was between 81–83%. As Steinborn, Flehmig, Bratzke, et al. [52] noted that individuals with higher accuracy exhibited larger post-error RT, suggesting that an orienting response phenomenon caused by infrequent events may account for our different findings. Moreover, Danielmeier and Ullsperger [10] proposed that PES is more obvious when using a shorter response-stimulus interval (RSI). Therefore, the prolonged PES that Themanson and Hillman [16] observed might be due to the shorter RSI in contrast with the current task design. It is noteworthy that the potential causal relationship between overall RT and PES could not account for our findings since there was no difference in Go RT across groups, thus the decreased post-error RT and insignificant PES in the sport groups reflected the acceleration in the error-related processing instead of faster overall RT among athletes.

In terms of the putative characteristics of athletes, our finding on PES might be linked to the tendency shaped by training experience as well. Typically, athletes pursue their goals by speeding up to maintain or ameliorate performance in competitions, which plays a role in modulating the reactions in the error-related process. For example, taekwondo athletes are well-known to adopt a fast speed to change strategies after realizing an improper plan was deployed and deal with an opponent’s attack in seconds, or swimmers correct every motion with a quick pace, whereas speed may not be necessarily important for non-athletes in their daily life. Meanwhile, the automaticity and psychological traits of athletes may provide another explanation with athletes having higher cognitive flexibility to induce greater effectiveness of the information- and decision-making process [53]. They took advantage of several strategies to achieve the optimal behavioral pattern with effortlessness, less consciousness, and regulate emotional states if peak performance is not met [54]. Therefore, if the athletes in the current study were exerting increased emotional regulation, it would make sense that the decreased PES among them because of the significant relationships between PES, negative affect, and coping with stress [55, 56].

There are a few advantages in the current study. First, we applied the protocol of stop signal delay to ensure the adequate number of stop errors which allows fewer trials in the cognitive assessment compared to the previous studies with similar interests. Therefore, presumably, participants could maintain attention in the task without much fatigue. Second, instead of recruiting individuals without structured sport training experience, we included athletes who are more representative of the populations with higher PA. However, this study also has several disadvantages. Above all, the cross-sectional design cannot draw the evidence of the causal relationship between sport training and altered cognition, thus we encourage that future studies examine this causality via randomized controlled trials. Second, since brain imaging and behavioral evidence linked fitness performance to altered brain structures [57], brain functional connectivity [58], and better executive function [59], the lack of fitness measurement limits the inference of our results which leaves a venue for future works to objectively estimate both cardiorespiratory fitness and physical activity to better understand the association between sport and inhibition and error processing. Lastly, this study may be limited by its sample size, which lowers the ability to detect differences in inhibition, although it is clear that we were able to report significant differences in PES.

In conclusion, we expanded previous findings of the differences in cognitive performance between individuals with and without long-term sport participation as well as between different types of athletes, especially for inhibition and error monitoring in a combined flanker/stop signal task. The results showed inhibitory control was insignificant between groups, nevertheless, measures of PES demonstrated attenuated PES for open- and close-skill sport groups as well as faster post-error responses for the open-skill individuals compared with sedentary controls. However, the post-error accuracy was not significantly different between groups, indicating a more efficient functioning of error detection and adjustment among open- and closed-skill groups. Therefore, the fundamental error-related subcomponent of cognitive control might benefit from the combination of higher PA and training in sport, particularly when the training involves open-skill in nature which is associated with abundant cognitive stimuli. These findings demonstrate important implications for the studies that focus on the populations with deficits in error processing such as individuals with substance abuse [60], attention-deficit/hyperactivity disorder (ADHD) [61], schizophrenia [62], depressive symptoms [63], or violent behaviors [64]. In addition to the structured and regular physical activity, sports that require open-skill may potentially provide a proper intervention for these clinical populations. Furthermore, taking into consideration of confounding factors, future research that explores the potential influences of mediator/moderator (e.g. emotional regulation, stress coping, and personality traits) on the relationships between sport and inhibition as well as error monitoring is needed. To give an insight into the differences in cognition between athletes and non-athletes, it may be of interest to adopt tasks with sport-related stimuli to test whether the benefits of sport are generalizable for common settings or selective for sport-specific settings.
